# Rapid and accurate detection of *Fusarium oxysporum* f. sp. Lycopersici using one-pot, one-step LAMP-CRISPR/Cas12b method

**DOI:** 10.3389/fpls.2024.1485884

**Published:** 2024-12-20

**Authors:** Shijie Dai, Yangsheng Wu, Na Zhu, Yujin Zhao, Mingjiang Mao, Zheming Li, Bo Zhu, Weichun Zhao, Xiaofeng Yuan

**Affiliations:** ^1^ College of Life Science, Zhejiang Chinese Medical University, Hangzhou, Zhejiang, China; ^2^ School of Pharmaceutical Sciences, Zhejiang Chinese Medical University, Hangzhou, China; ^3^ Academy of Chinese Medical Sciences, Zhejiang Chinese Medical University, Hangzhou, Zhejiang, China

**Keywords:** FOL, LAMP, Cas12b, one-pot, point-of-need, phytopathogen detection

## Abstract

**Introduction:**

*Fusarium oxysporum* f. sp. *Lycopersici* (Fol) is one of the most devastating plant pathogenic fungi, the causal agent of root rot for *A*tractylides macrocephala Koidz (AMK). An accurate rapid and convenient diagnosis for FoL detection is essential for determining management practices and preventing future losses for AMK.

**Methods:**

Here, we developed a novel method for Fol detection by integrating loop-mediated isothermal amplification (LAMP) assay and CRISPR/Cas12b detection in one-pot, and the whole reaction can simultaneously amplify and detect the target gene of Fol in one-step.

**Results:**

The total time of the present method is limited to 45 min and isothermally performed at 60°C. The limit of detection of this assay is 88.9 copies per reaction. The specificity of the LAMP-CRISPR/Cas12b method was 100% without any cross-reaction of other pathogens. A total of 24 nucleic acid samples were used to evaluate the performance of the LAMP-CRISPR/Cas12b method, including 12 with-Fol and 12 without-Fol. Compared with the gold standard results from real-time PCR, the present method provides a sensitivity of 100% (12/12), specificity of 100% (12/12), and consistency of 100% (24/24).

**Discussion:**

Together, our preliminary results illustrated that the LAMP-CRISPR/Cas12b method is a rapid simple, and reliable tool for Fol diagnosis and could be applied in point-of-need phytopathogen detection.

## 1 Introduction

*Atractylodes macrocephal*a Koidz (AMK) is one of the well-known perennial herbs, which is widely used in traditional Chinese medicine (Zhu et al., 2020). It has been confirmed that AMK exhibits super pharmacological effects in alleviating spleen deficiency, abdominal distension, diarrhea, and constipation (Chen et al., 2024). Moreover, recent evidence has demonstrated that AMK extract ameliorates insulin resistance, Type-2 diabetes mellitus, cancer, osteoporosis, gastrointestinal dysfunction, obesity, and fetal irritability (Li et al., 2024; Zhu et al., 2018). Commonly, the AMK is predominantly distributed in mountainous wetlands in China, such as Zhejiang, Sichuan, Yunnan, and Hunan provinces. As a perennial herb, the medicinal production and quality are deeply disrupted by root rot disease (Gao et al., 2024). To maintain a stable production of AMK, a timely and accurate diagnosis method for root rot disease identification is critically important.

*Fusarium oxysporum* f. sp. *Lycopersici* (Fol) is the major cause of Fusarium crown and root rot, which leads to considerable field yield losses all over the world (Swett et al., 2023; Nirmaladevi et al., 2016; Yue et al., 2022). Fol is a soil-borne phytopathogenic species that penetrates plant roots before colonizing the vascular tissue, which can survive in field soil for several years in the form of chlamydospores and can spread via infested soil on equipment or infected seed. It is well known that the early detection of plant diseases is crucial because it is the key to effective disease control, especially for most soilborne pathogens, once symptoms have developed in a crop, it is already too late for disease management to occur (Testen et al., 2021; Bakker et al., 2020). To minimize the root rot-caused impact on AMK, a rapid and reliable method for the earlier detection of Fol is important.

The traditional method for plant fungal disease identification has relied on field inspections and laboratory culture-based tests (Takken and Rep, 2010). However, these plant funguses are easily misjudged by the similar morphology and the whole detection was time-consuming, laborious, and experienced operator. As a novel traditional method, the Polymerase chain reaction (PCR)-based method has become a routine assay for pathogens detection and has been used for Fol detection (Del Mar Jiménez-Gasco and Jiménez-Díaz, 2003; Larena and Melgarejo, 2009). However, this approach was restricted by the requirement of high-cost equipment, high-skilled technicians, and time-consuming. Compared with traditional methods, isothermal amplification can be performed at a constant temperature without additional requirements as that of PCR, such as loop-mediated isothermal amplification (LAMP) and recombinase polymerase amplification (RPA) assay, For example, LAMP-based platforms have been discovered and used to detect and differentiate Fol and presented outstanding advantages in sensitivity (Ayukawa et al., 2016, 2017). However, the false-positive results led by the non-specific products of isothermal-based assays remain higher and not well-addressed (Zhang et al., 2021; Bhat et al., 2022; Yang et al., 2024).

Clustered regularly interspaced short palindromic repeat (CRISPR)-associated endonuclease (CRISPR/Cas) system is a novel nucleic acid-based detection platform and has been applied for plant disease diagnosis (Islam and Kasfy, 2023; Sharma et al., 2021; Chen et al., 2018; Li et al., 2018, 2019). In this system, the target nucleic acid was amplified using isothermal amplification techniques and then detected by CRISPR/Cas system based on their trans-cleavage activity. Once the ternary complex with Cas proteins, sgRNA, and target sequence was formed, the trans-cleavage activity of Cas proteins was activated and will extensively cleave non-target single-stranded nucleic acids in the system. By using the fluorophore quencher-labeled single-stranded nucleic acid reporter, the target sequence can be detected by monitoring the fluorescent signal of the reaction system. As the Cas protein was extremely sensitive to the mismatch and could not tolerate even one single base. Therefore, the type of this platform was highly specific. Moreover, a preamplification via isothermal amplification assay was commonly suggested to combine with CRISPR/Cas detection system and the corresponding platforms have been established for detecting *Bursaphelenchus xylophilus* (Wang et al., 2022) and Xanthomonas arboricola (Luo et al., 2021). However, the operation of product transfer to the CRISPR/Cas detection system posed a risk of aerosol cross-contamination (Hu et al., 2022). For this issue, several one-pot CRISPR-based platforms have been established for identifying *Diaporthe aspalathi* (Dong et al., 2024) and plant RNA Viruses (Aman et al., 2020), which was achieved by adding components at the bottom and wall of the tube separately. Therefore, the operation was skilled and not easy for fresh users. However, the corresponding system for detecting Fol-caused root rot in AMK remains unknown.

In this study, we first developed a one-step Fol detection system by combining the LAMP assay with CRISPR/Cas12b in one-pot. The whole assay can be finished within 45 min at a constant temperature of 60°C without additional procedures, achieving a rapid and contamination-free advantage. Moreover, the present assay was specific for Fol, and no-cross reaction was identified. Therefore, our present one-step LAMP CRISPR/Cas12b detection system was a promising platform for Fol diagnosis.

## 2 Material and methods

### 2.1 Reagents

The LAMP nuclear amplification kit was obtained from TOLO Biotech (25102, Shanghai, China), AapCas12b was purchased from TOLO Biotech (32118, Shanghai, China), HOLMES ssDNA reporter (FAM) was provided from TOLO Biotech (31101, Shanghai, China), Cas12b High Yield sgRNA Synthesis and Purification Kit was purchased from TOLO Biotech (31904, Shanghai, China), PCR Mixture was purchased from ZHENZHUN BIO (MX0108, Shanghai, China). Nuclease-free water was purchased from Solarbio Life Sciences (R1600, Beijing, China).

### 2.2 Sample preparation

The Fol strain used in the present study was collected from the American Type Culture Collection (NO.bio-72843). The commercial DNA isolation kit (TIANGEN, Beijing, China) was used to extract the genomic DNA of Fol according to the instructions of the manufacturer. To generate the recombinant plasmid pUC57-Fol_SIX1, the target gene was inserted into the pUC57 vector. The concentration of the recombinant plasmid was 44.1 ng/μL, which corresponds to a copy number of 532.1 copies/μL, then used as a standard product for the LAMP-CRISPR/Cas12b detection system.

### 2.3 Primer and sgRNA design

According to the conserved region of Fol gene *SIX1* (GenBank: U66901.1), the LAMP primer was designed by using the Online NEB LAMP primer design tool (https://lamp.neb.com/#!/). The Cas12b sgRNA was designed based on the amplified sequence of *SIX1*. All the primers used in the present study were synthesized in Sangon Biotech (Shanghai, China). The Cas12b sgRNA was purified by using the Cas12b High Yield sgRNA Synthesis and Purification Kit (31904, ToloBio, China) according to the instruction of the manufacturer.

### 2.4 LAMP reaction

The total volume of the LAMP reaction 25 µL, the mixture includes 2.5 µL of 10 x LAMP isothermal reaction buffer, 1.4 µL dNTP mix (25 mM), 1.75 µL MgSO_4_ (100 mM), 2.5 µL 10× Fol_SIXI1 LAMP primer mix, 0.25 µL 100×SYTO-9, 1 µL of Bst 2.0 DNA polymerase, 2.5 µL Template, and Nuclease-free water up to 25 µL. LAMP amplification was performed at 60°C for 30 min. The pUC57- Fol_SIXI1 plasmid was used as the template, while nuclease-free water served as the no-template control (NTC, negative control). Three repeats were needed for each reaction The LAMP assay was performed on the Applied Biosystems QuantStudio 5 Real-Time PCR System (QuantStudio 5, ThermoFisher, USA).

### 2.5 Establishment and optimization of one-step LAMP-CRISPR/Cas12b reaction condition

The one-step LAMP-CRISPR/Cas12b assay was established and optimized as follows, the total volume of Fol-LAMP-CRISPR/Cas12b reaction mixture was 25 µL including 2.5 µL 10× LAMP Fluorescent buffer (100 rxns), 0.8/1.0/1.2/1.4 µL dNTP mix (25 mM), 1.5/1.75/2/2.25/2.5 µL MgSO_4_ (100 mM), 2.5 µL 10 × Fol_SIXI1 LAMP primer mix, 1.25 µL HOLMES ssDNA reporter (10 μM), 0.1563/0.3125/0.625/1.25 AapCas12b (10 μM), 1µL Bst (8 U/μL), 0.1563/0.3125/0.625/1.25 Fol_SIXI1-sgRNA (10 μM), 2.5 µL Template. Then, Nuclease-free water was used to make up to 25 µL. The reaction was performed at 60°C for 45 min on the Applied Biosystems QuantStudio 5 Real-Time PCR System (QuantStudio 5, ThermoFisher, USA), and the fluorescent accumulation was collected every 30 s. Three repeats were needed for each reaction.

### 2.6 Validation of the quantitative real-time PCR

The genomics DNA of the Fol-infected AMK from Pot cultivation experiments and field experiments was extracted as previously described. Then, qPCR was conducted in a 20 µl reaction volume containing 4 μL 5× PCR buffer, 0.16 μL dNTP mix (25 mM), 0.2 μL Taq DNA Polymerase (2.5 U/μL), 0.6 μL Fol_SIX1-F1 (5’-TT GGGTTTGGGGCTTATGCT-3’), 0.6 μL Fol_SIX1-R1 (5’-ACCATCGTCCGT CTC GTTTC-3’), 0.2 μL 100× SYTO-9, 12.24 μL Nuclease-free water, and 2 μL Template using a QuantStudio 5 Real-Time PCR System (QuantStudio 5, ThermoFisher, USA). Thermal cycling was carried out with the following conditions: 95 °C for 2 min, followed by 40 cycles of 95°C for 10 s, 78°C for 30 s, and 95°C for 15 s, and a final extension at 60°C for 10 min.

## 3 Results

### 3.1 The principle and workflow of LAMP-CRISPR/Cas12b detection system for Fol

**Figure 1:**
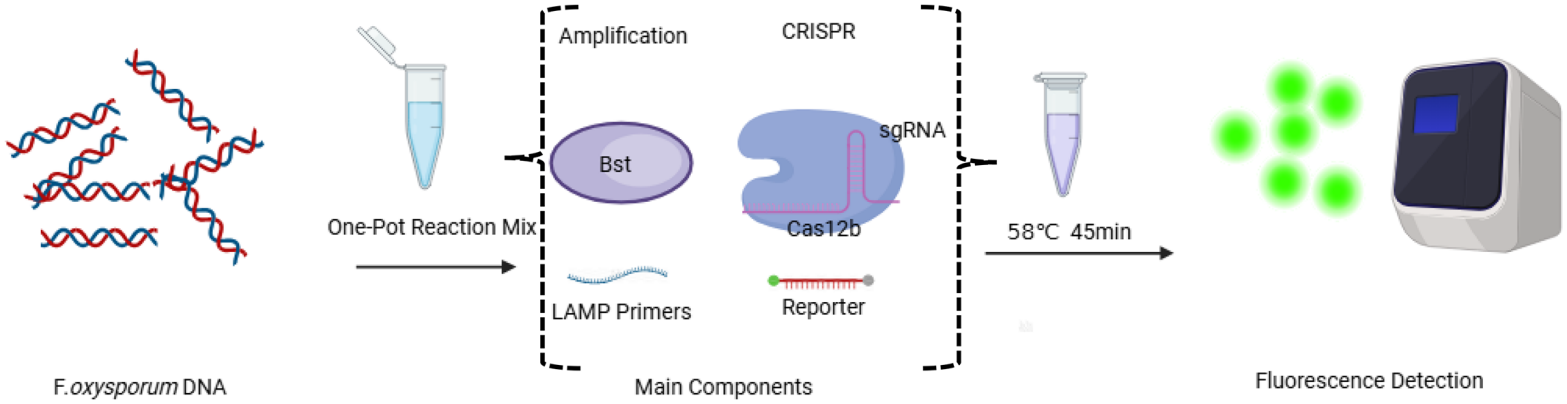
Schema illustration of the workflow for Fol one-step LAMP CRISPR/Cas12b detection system.

The principle and operation of Fol detection integrated with the LAMP and CRISPR/Cas12b system are shown in **Figure 1**. In summary, the crude genomic DNA of Fol was extracted and subsequently transferred to the LAMP-CRISPR/Cas12 detection platform. The whole reaction was performed in a single reaction step at a constant temperature for nucleic acid detection. The LAMP-amplified products can be recognized by the corresponding Cas12b/sgRNA system, then the trans-cleavage of Cas12b was activated and then cleaved the fluorophore 6-FAM and quencher BHQ1-labeled reporter DNA resulted in the appearance of fluorescence, then the results can then be detected on real-time PCR fluorescence readout or the other small and portable blue light instruments. The whole test can be finished within 1 h, including 15 min rapid template preparation and 45 min LAMP-CRISPR/Cas12b detection.

### 3.2 Selection of LAMP primer for one-step LAMP-CRISPR/Cas12b reaction

**Figure 2:**
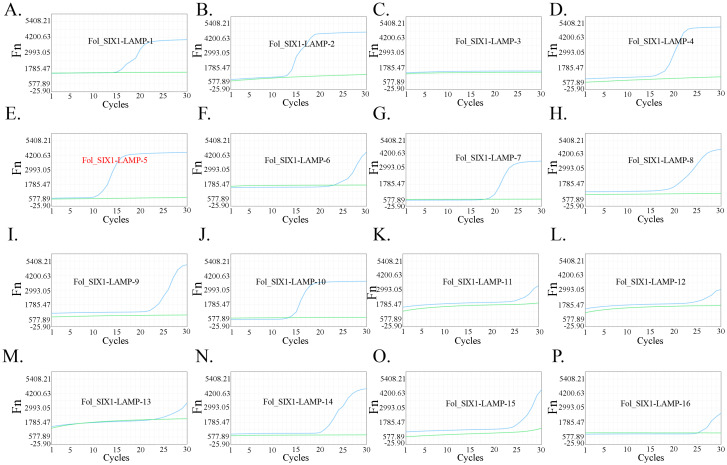
Selection of the LAMP primers for Fol one-step LAMP CRISPR/Cas12b detection system. The selection results of sixteen groups LAMP primer were shown from **(A–P)** respectively. Fol_SIXI_LAMP5 **(E)** exhibited a better performance than that of others.

SIX1 is identified as a Fol species-specific protein, which is chosen as the target for detection (Rep et al., 2004). Therefore, SIXI was chosen as the target gene for Fol identification in the present study. Moreover, the specific conserved sequence of SIX1 was inserted into a recombinant plasmid. Then, a total of 16 LAMP primers (Fol_SIX1-LAMP-1 to Fol_SIX1-LAMP-16, seen in **Table 1**) were designed. The recombinant plasmid was used as the template. The reaction by using Fol_SIX1-LAMP-5 primers presented the earliest take-off time than that of other LAMP primers (**Figures 2A–P**), thereby Fol_SIX1-LAMP-5 was chosen for target sequence amplification.

**Table 1:** The LAMP primer used in the present study.

LAMP Primer		Sequence (5’-3’)
Fol_SIX1-LAMP-1	Fol-F3-1	TCGGTCTGGACACCATCG
	Fol-B3-1	TCCTTGGGTTTGGGGCTTA
	Fol-FIP-1	ACGGGAGTCCCCCAGACAAGTCCGTCTCGTTTCCAGGAA
	Fol-BIP-1	GGATGCTGCCACCTTATCCAGACTCAAGAGGCTGCGGTTC
	Fol-LF-1	GCCCTGGAATGACACTATGGG
	Fol-LB-1	CAAGTTGAAAAATATCTGGGGCTCC
Fol_SIX1-LAMP-2	Fol-F3-2	TGTGACCACGCCTCGATAG
	Fol-B3-2	TCCTTGGGTTTGGGGCTTA
	Fol-FIP-2	GCCCTGGAATGACACTATGGGCGTCTCGGTCTGGACACCA
	Fol-BIP-2	TGGGATGCTGCCACCTTATCCACTCAAGAGGCTGCGGTTC
	Fol-LF-2	CCTGGAAACGAGACGGACGA
	Fol-LB-2	CAAGTTGAAAAATATCTGGGGCTCC
Fol_SIX1-LAMP-3	Fol-F3-3	TGTGTGACACGATCGTTGC
	Fol-B3-3	TCCCCCAGACAAGAGTGATT
	Fol-FIP-3	CCTATCGAGGCGTGGTCACATTGAGACTCCTCCCCGAAAGG
	Fol-BIP-3	TCTCGGTCTGGACACCATCGTGCCCTGGAATGACACTATGG
	Fol-LB-3	CGTCTCGTTTCCAGGAAAGCTG
Fol_SIX1-LAMP-4	Fol-F3-4	CTGATGGTGACGGTTACGAA
	Fol-B3-4	CAGGCGCTTCATGATCTGT
	Fol-FIP-4	CCGTCTGTCAGGGAGAGAGACAGTACGCATTGAAAGTGGTGC
	Fol-BIP-4	CGATTTTCCTAGTCGCCCGACGGCGTTGAAAGATTTGGCACA
	Fol-LF-4	TGCCCAAAGGGCCAGGA
	Fol-LB-4	GTGTAACATAACCCACTAACCGCTC
Fol_SIX1-LAMP-5	Fol-F3-5	GAAAGTGGTGCACTCCTGG
	Fol-B3-5	GTCTCGCAACGATCGTGTC
	Fol-FIP-5	CGTCGGGCGACTAGGAAAATCGCCTTTGGGCAAGCTCTGG
	Fol-BIP-5	AACCCACTAACCGCTCGATAGCACACAAGACATGCTCCAGG
	Fol-LF-5	CAGGGAGAGAGACAGATCAGTGA
	Fol-LB-5	CTGTGCCAAATCTTTCAACGCA
Fol_SIX1-LAMP-6	Fol-F3-6	CTTTGGGCAAGCTCTGGT
	Fol-B3-6	GGAGGAGTCTCGCAACGA
	Fol-FIP-6	ACACTGATCGTCGGGCGACTAGTGTCTCTCTCCCTGACAGAC
	Fol-BIP-6	AACCCACTAACCGCTCGATAGCTCACACAAGACATGCTCCAG
	Fol-LB-6	CTGTGCCAAATCTTTCAACGCA
Fol_SIX1-LAMP-7	Fol-F3-7	GATAGTCACCTTGCCTCTCG
	Fol-B3-7	CCCCGAATTGAGGTGAAGG
	Fol-FIP-7	TGGGTACTTCGGGTATGACGGTTGCATGACCATGAATGTCCA
	Fol-BIP-7	CGTAGGTCCCAGGCGATTTAGGAGGGATACACACGGAGTGG
	Fol-LF-7	GGATATAAGACGAGCTCTCACGGAT
Fol_SIX1-LAMP-8	Fol-F3-8	TCCGTGAGAGCTCGTCTT
	Fol-B3-8	CTTTTCCTGTCTGTGGGCC
	Fol-FIP-8	CCTAAATCGCCTGGGACCTACGAACACCGTCATACCCGAAGT
	Fol-BIP-8	CGATTCGGGGTACCAGGTTCCCCGAATTGAGGTGAAGGACA
	Fol-LF-8	ATGGCGGGCACTCTCAATG
	Fol-LB-8	GCCACTCCGTGTGTATCCCTAT
Fol_SIX1-LAMP-9	Fol-F3-9	GGAGTCCCCCAGACAAGAG
	Fol-B3-9	TGTGACACGATCGTTGCG
	Fol-FIP-9	GGACACCATCGTCCGTCTCGTTGATTTGCCCTGGAATGACA
	Fol-BIP-9	CAGCAGCCTATCGAGGCGTGAGACTCCTCCCCGAAAGG
	Fol-LF-9	TTCCAGGAAAGCTGCCCATAG
	Fol-LB-9	TCACATTGTCAACCTTAGAAAGCGT
Fol_SIX1-LAMP-10	Fol-F3-10	GGAGTCCCCCAGACAAGA
	Fol-B3-10	GCCAAATCTTTCAACGCACA
	Fol-FIP-10	GATAGGCTGCTGCCCGTCTCGACACTATGGGCAGCTTTCC
	Fol-BIP-10	CGTGAGCCTTTCGGGGAGGACGCCTGGAGCATGTCTTG
	Fol-LF-10	CCATCGTCCGTCTCGTTTCC
	Fol-LB-10	TCTCGCAACGATCGTGTCACA
Fol_SIX1-LAMP-11	Fol-F3-11	TGGAATGACACTATGGGCAG
	Fol-B3-11	ACGCACAGATCATGAAGCG
	Fol-FIP-11	GACCACGCCTCGATAGGCTGTGGAAACGAGACGGACGA
	Fol-BIP-11	TAGAAAGCGTGAGCCTTTCGGGGGAGCATGTCTTGTGTGACA
	Fol-LF-11	CGTCTCGGTCTGGACACCA
Fol_SIX1-LAMP-12	Fol-F3-12	GCCCTGGAATGACACTATGG
	Fol-B3-12	GCCAAATCTTTCAACGCACA
	Fol-FIP-12	GATAGGCTGCTGCCCGTCTCGCAGCTTTCCTGGAAACGA
	Fol-BIP-12	TAGAAAGCGTGAGCCTTTCGGGGGAGCATGTCTTGTGTGACA
	Fol-LF-12	TCTGGACACCATCGTCCGT
Fol_SIX1-LAMP-13	Fol-F3-13	CTATGGGCAGCTTTCCTGG
	Fol-B3-13	GCCAAATCTTTCAACGCACA
	Fol-FIP-13	GGTTGACAATGTGACCACGCCTCGGACGATGGTGTCCAGA
	Fol-BIP-13	TAGAAAGCGTGAGCCTTTCGGGGGAGCATGTCTTGTGTGACA
	Fol-LF-13	TAGGCTGCTGCCCGTCT
Fol_SIX1-LAMP-14	Fol-F3-14	GATCATGAAGCGCCTGGAG
	Fol-B3-14	CAAGAGTGATTTGCCCTGGA
	Fol-FIP-14	GGCGTGGTCACATTGTCAACCTTTGTGTGACACGATCGTTGC
	Fol-BIP-14	GATAGGCTGCTGCCCGTCTCACACTATGGGCAGCTTTCCT
	Fol-LF-14	TGAGCCTTTCGGGGAGGAG
	Fol-LB-14	GTCTGGACACCATCGTCCGT
Fol_SIX1 LAMP-15	Fol-F3-15	GCCAAATCTTTCAACGCACA
	Fol-B3-15	CACTATGGGCAGCTTTCCTG
	Fol-FIP-15	CGTGAGCCTTTCGGGGAGGACGCCTGGAGCATGTCTTG
	Fol-BIP-15	AGGTTGACAATGTGACCACGCCGAAACGAGACGGACGATGG
	Fol-LF-15	CTCGCAACGATCGTGTCACA
Fol_SIX1-LAMP-16	Fol-F3-16	CCTGGAGCATGTCTTGTGT
	Fol-B3-16	CAAGAGTGATTTGCCCTGGA
	Fol-FIP-16	GGCGTGGTCACATTGTCAACCTCGATCGTTGCGAGACTCCT
	Fol-BIP-16	GATAGGCTGCTGCCCGTCTCACACTATGGGCAGCTTTCCT
	Fol-LB-16	CACCATCGTCCGTCTCGTTTC

### 3.3 Selection of sgRNA for one-step LAMP-CRISPR/Cas12b reaction

**Figure 3:**
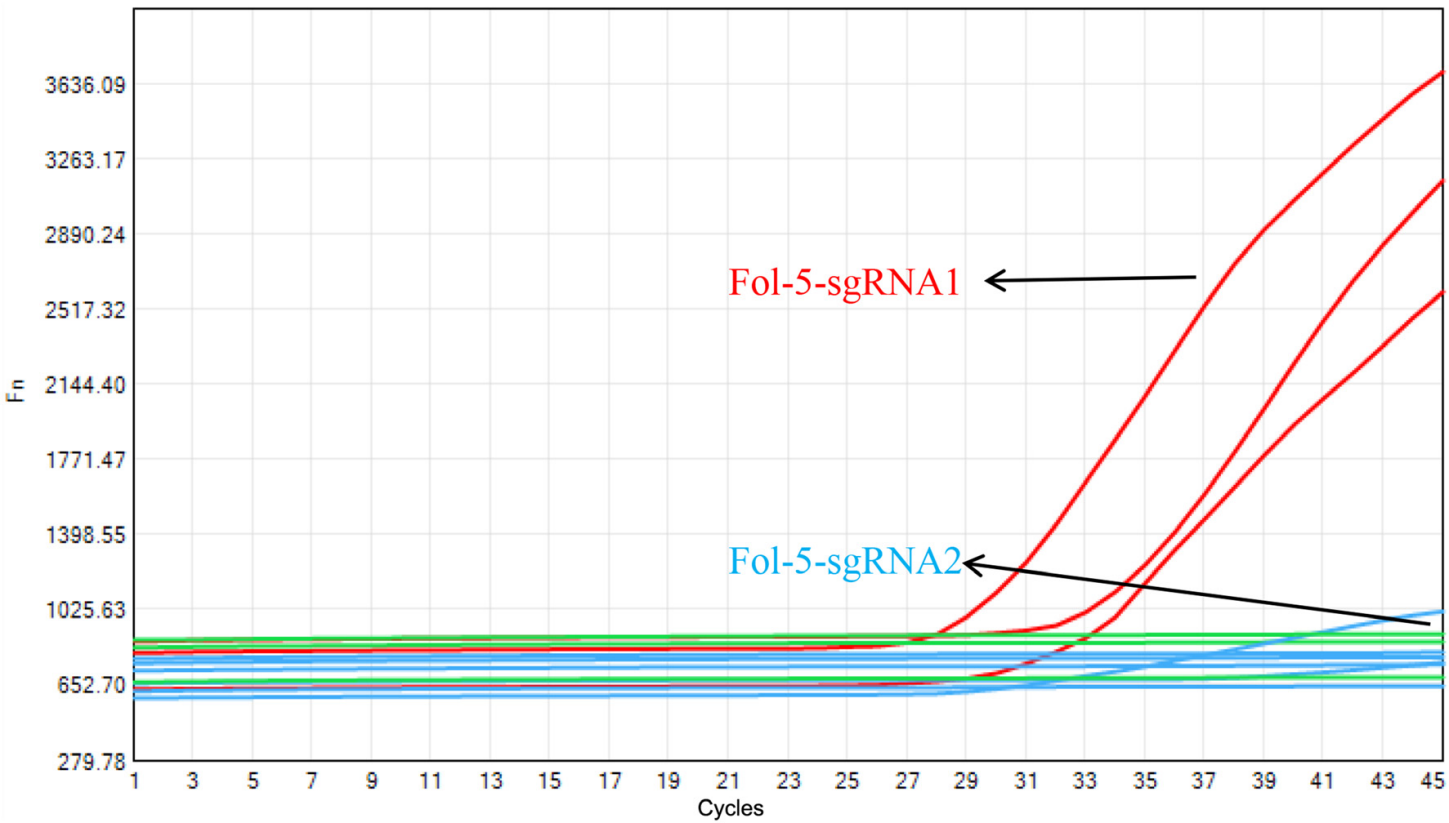
Selection of the Cas12b sgRNA for Fol one-step LAMP CRISPR/Cas12b detection system. Fol-5-sgRNA1 presented a better property than that of Fol-5-sgRNA2.

Based on the amplification product of Fol_SIX1-LAMP-5, two specific sgRNAs were designed and synthesized, named Fol-5-sgRNA-1 and Fol-5-sgRNA-2, as seen in **Table 2**. Taking the take-off time and fluorescent intensity into consideration, Our data suggested that Fol-5-sgRNA-1 showed a better performance than that of Fol-5-sgRNA-2 (**Figure 3**). Therefore, Fol-5-sgRNA-1 was used for target recognition in one-step Fol detection system.

**Table 2:** The sgRNA used in the present study.

sgRNA	Sequences(5’-3’)
Fol-5-sgRNA1	GUCUAGAGGACAGAAUUUUUCAACGGGUGUGCCAAUGGCCACUUUCCAGGUGGCAAAGCCCGUUGAGCUUCUCAAAUC UGAGAAGUGGCACGACAGACGGUUUAUUACAUU
Fol-5-sgRNA2	GUCUAGAGGACAGAAUUUUUCAACGGGUGUGCCAAUGGCCACUUUCCAGGUGGCAAAGCCCGUUGAGCUUCUCAAAUC UGAGAAGUGGCACACAGACGGUUUAUUACAUUC

The target sequences were underlined.

#### 3.3.1 Optimal dNTP and MgSO_4_ concentration

**Figure 4:**
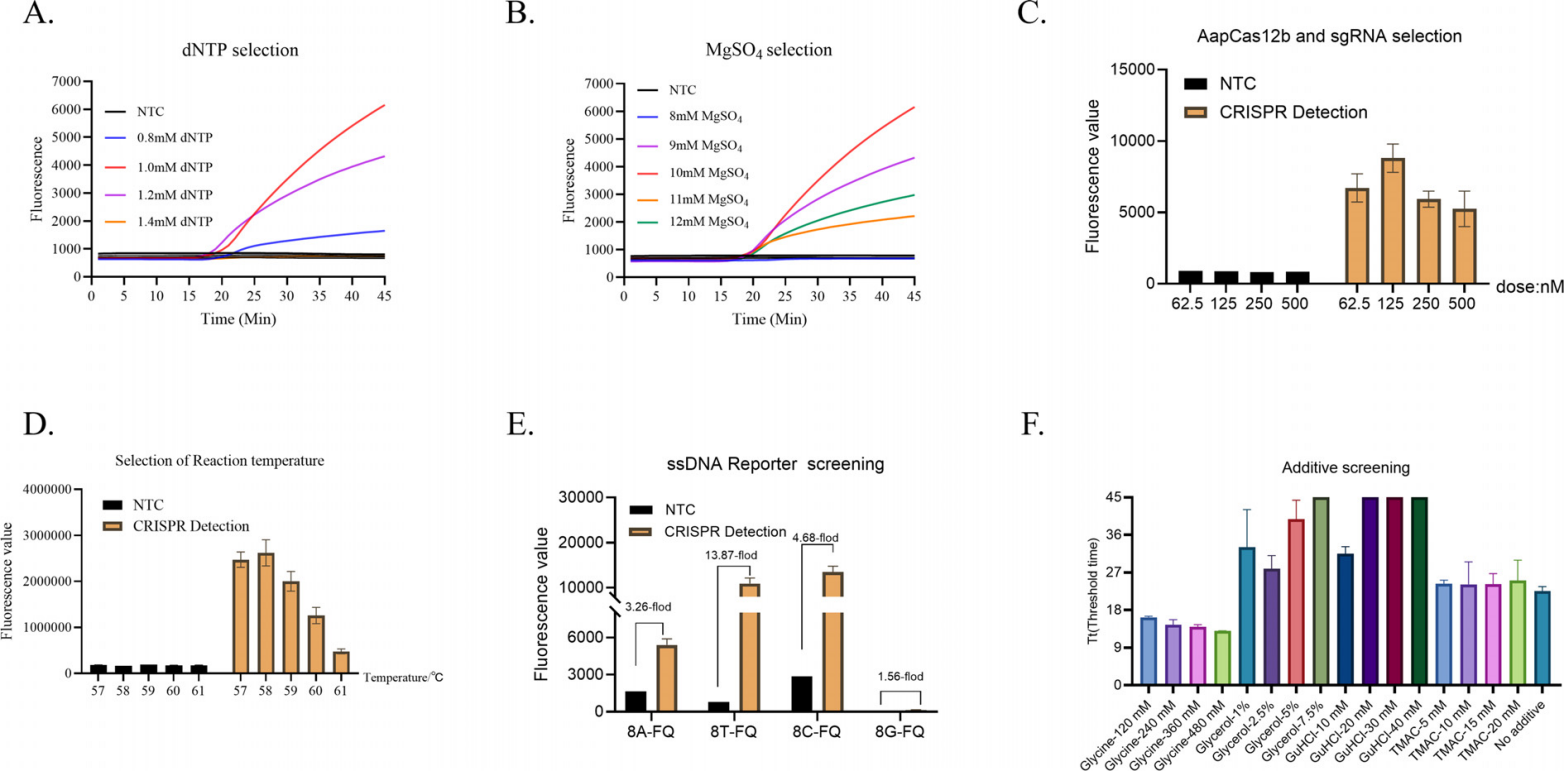
Optimal conditions for one-step LAMP-CRISPR/Cas12b method. Optimization of dNTP **(A)** and Mg^2+^ **(B)**, Cas12b and sgRNA **(C)**, reaction temperature **(D)**, ssDNA reporter **(E)**, and additives **(F)**.

To determine the optimal dose of dNTP for one-step LAMP CRISPR/Cas12b assay, the dNTP was diluted into 0.8 mM, 1.0 mM, 1.2 mM, and 1.4 mM respectively, corresponding to the volume of 0.8 µL, 1.0 µL, 1.2 µL, and 1.4 µL in the reaction. As shown in **Figure 4A**, the fluorescence intensity of the reaction reached the highest by using the dNTP in 1.0 mM. Therefore, the dose of dNTP was optimized at 1.0 mM. Then, the MgSO_4_ was diluted into 8 mM, 9 mM, 10 mM, 11 mM, and 12 mM respectively, corresponding to the volume of 1.5 µL, 1.75 µL, 2.0 µL, 2.25 µL and 2.5 µL in the reaction. As shown in **Figure 4B**, the fluorescence intensity of the 10 mM MgSO_4_ reaction was higher than that of other concentrations. Therefore, the dose of MgSO_4_ was optimized in 10 mM for one-step LAMP CRISPR/Cas12b Fol detection system.

#### 3.3.2 Optimal the concentrations of Cas12b protein and sgRNA

To determine the optimal concentrations of Cas12b protein and Cas12b sgRNA, both the Cas12b protein and Cas12b sgRNA were paired (1:1) diluted into 62.5 nM, 125 nM, 250 nM, and 500 nM respectively. As shown in **Figure 4C**, the optimum reaction concentrations of Cas12b protein and Cas12b sgRNA in the dose of 125 nM, as it produced the highest fluorescence intensity.

#### 3.3.3 Optimal reaction temperature

Then, the optimal reaction temperature for the reaction was evaluated. The reaction temperature was set from 57°C to 61°C with 1°C increments. Clearly, the reaction condition at the temperature of 58°C showed the highest fluorescence intensity than that of other temperatures (**Figure 4D**). Hence, the one-step LAMP-CRISPR/Cas12b reaction condition at 58°C showed the highest fluorescence intensity compared to other temperatures.

#### 3.3.4 Optimal ssDNA reporter

Four different ssDNA reporters were used for selection, including 8A-FQ (5’-/6-FAM/AAAAAAAA/BHQ1/-3’), 8T-FQ (5’-/6-FAM/TTTTTTTT/BHQ1/-3’), 8C-FQ (5’-/6-FAM/CCCCCCCC/BHQ1/-3’), and 8G-FQ (5’-/6-FAM/GGGGGGGG/BHQ1/-3’). NTC was set for each reaction. As shown in **Figure 4E**, the ssDNA reporters of 8A-FQ, 8T-FQ, and 8C-FQ were able to induce a remarkable fluorescence intensity, while the 8G-FQ was not well-functioned. Taking the fluorescence intensity into consideration, the fold-changes achieved the highest by using an 8T-FQ reporter between NTC and CRISPR detection. Therefore, 8T-FQ was used for detection.

#### 3.3.5 Optimal additive for one-step LAMP-CRISPR/Cas12b reaction

Then, four different additives were used to select the best concentrations for the reaction, including Glycine (120 mM, 240 mM, 360 mM, and 480 mM), Glycerol (1%, 2.5%, 5%, and 7.5%), GuHCl (10 mM, 20 mM, 30 mM, and 40 mM), and TMAC (5 mM, 10 mM, 15 mM, and 20 mM) respectively. As shown in **Figure 4F**, the threshold time of the reaction showed no significant difference by using TMAC in different doses. Moreover, the threshold time of the reaction was increased by using Glycerol and GuHCL respectively. Only the additive of Glycine was able to reduce the threshold time and the take-off reached earliest by using Glycine in the dose of 480 mM.

### 3.4 Sensitivity of the Fol one-step LAMP-CRISPR/Cas12b method

**Figure 5:**
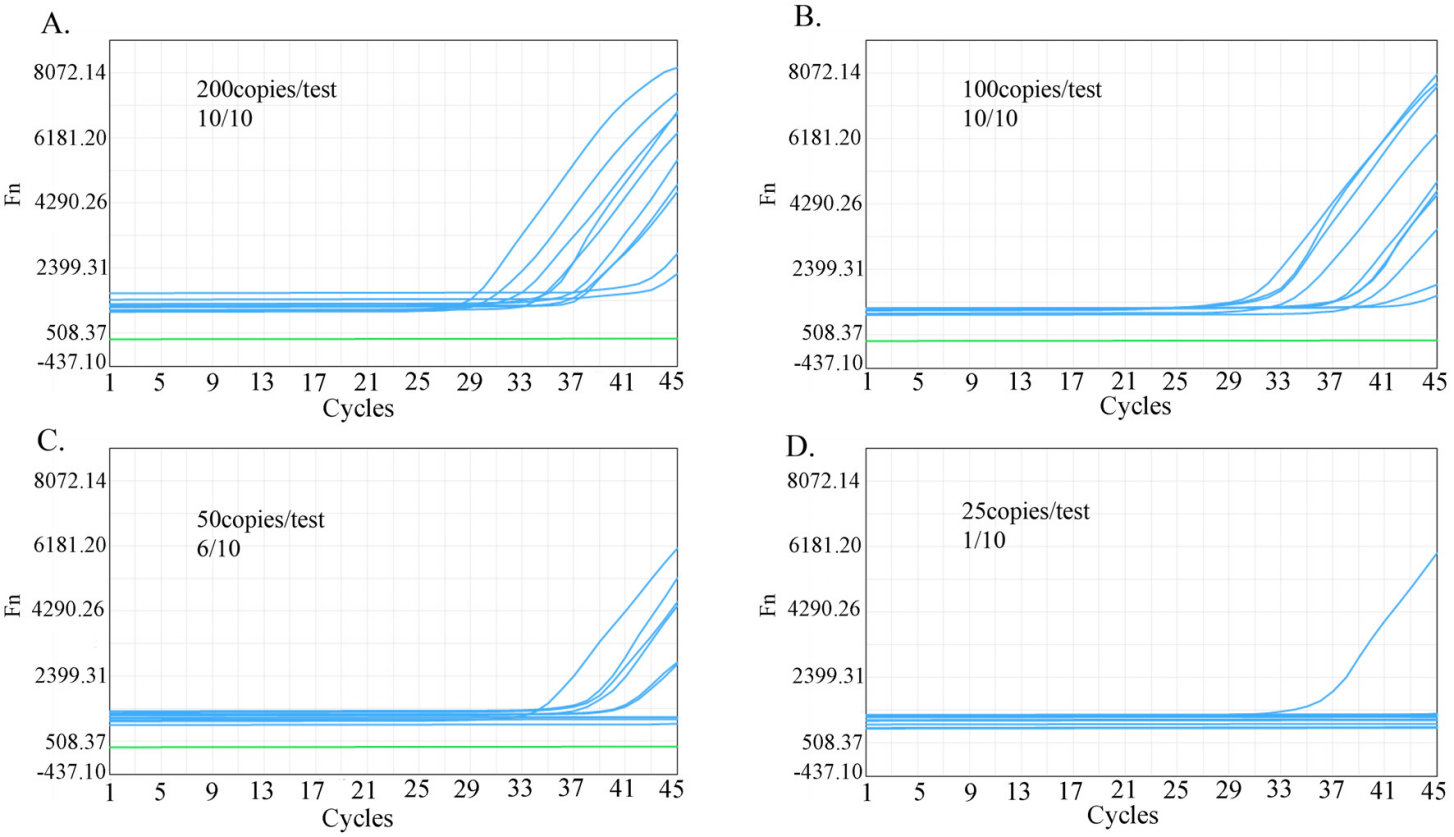
Sensitivity analysis of Fol one-step LAMP-CRISPR/Cas12b method. The positive reaction of the reaction that using the template in the dose of 200 copies/test **(A)**, 100 copies/test **(B)**, 50 copies/test **(C)**, and 25 copies/test **(D)**.

**Figure 6:**
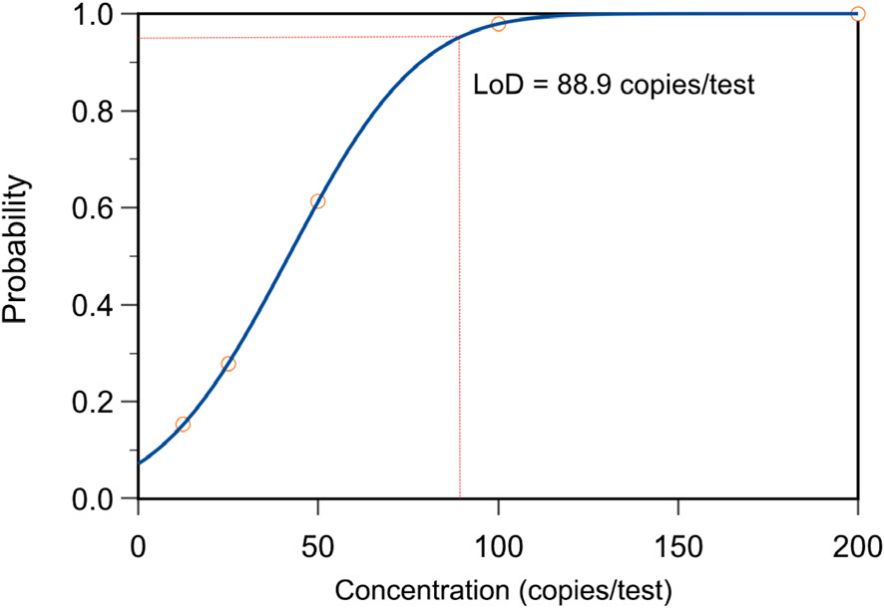
The limit of detection (LoD) analysis of Fol one-step LAMP-CRISPR/Cas12b method.

To determine the LOD of the Fol one-step LAMP-CRISPR/Cas12b assay, the recombinant plasmid (532.1 copies/μL) was diluted into 25 copies/reaction, 50 copies/reaction, 100 copies/reaction, and 200 copies/reaction. All ten replications were successfully identified by using the template in the dose of 100 copies/test and 200 copies/test, while the corresponding number was 6 and 1 for 50 copies/test and 25 copies/test respectively (**Figures 5A–D**). Based on these data, the limit of detection (LoD) of the present assay was predicted in 88.9 copies per reaction at the probability of 95% (**Figure 6**).

### 3.5 Specificity of the Fol one-step LAMP-CRISPR/Cas12b method

**Figure 7:**
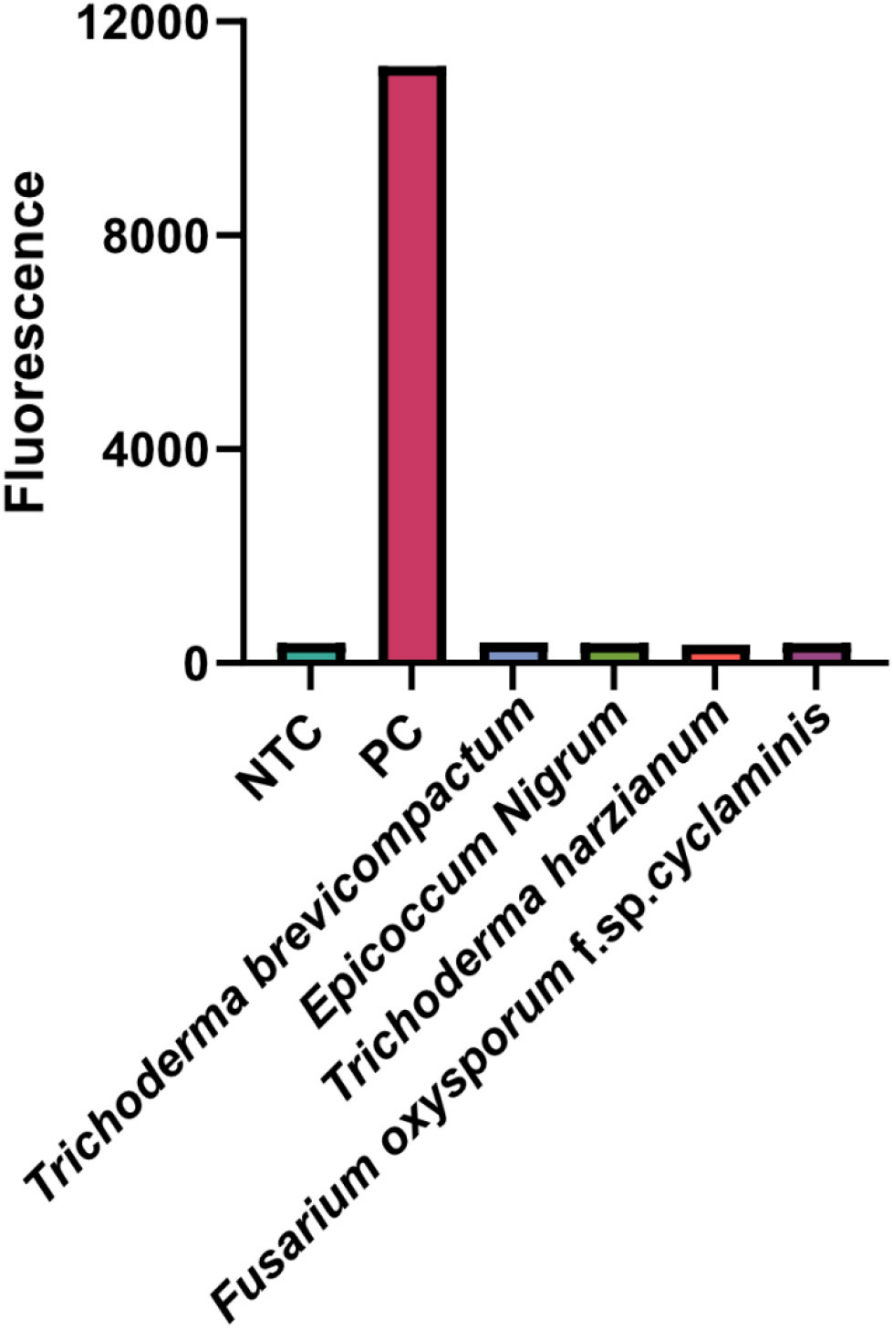
Specificity analysis of Fol one-step LAMP-CRISPR/Cas12b method.

To determine the specificity of the present reaction, four interfere nuclear acid samples were collected for detection, including *Trichoderma brevicompactum, Epicoccum Nigrum, Trichoderma harzianum, and Fusarium oxysporum f.sp.cyclaminis).* The recombinant plasmid was served as the positive control (PC), while the nuclease-free water used for NTC. As shown in **Figure 7**, only the PC reaction showed a remarkable fluorescence signal and the fluorescence signal of all interfere nuclear acid samples was equivalent to that of NTC. These results indicate that the present method is highly specific for Fol.

### 3.6 Validation the performance of the one-step LAMP-CRISPR/Cas12b system by using Fol-infected AMK samples

**Figure 8:**
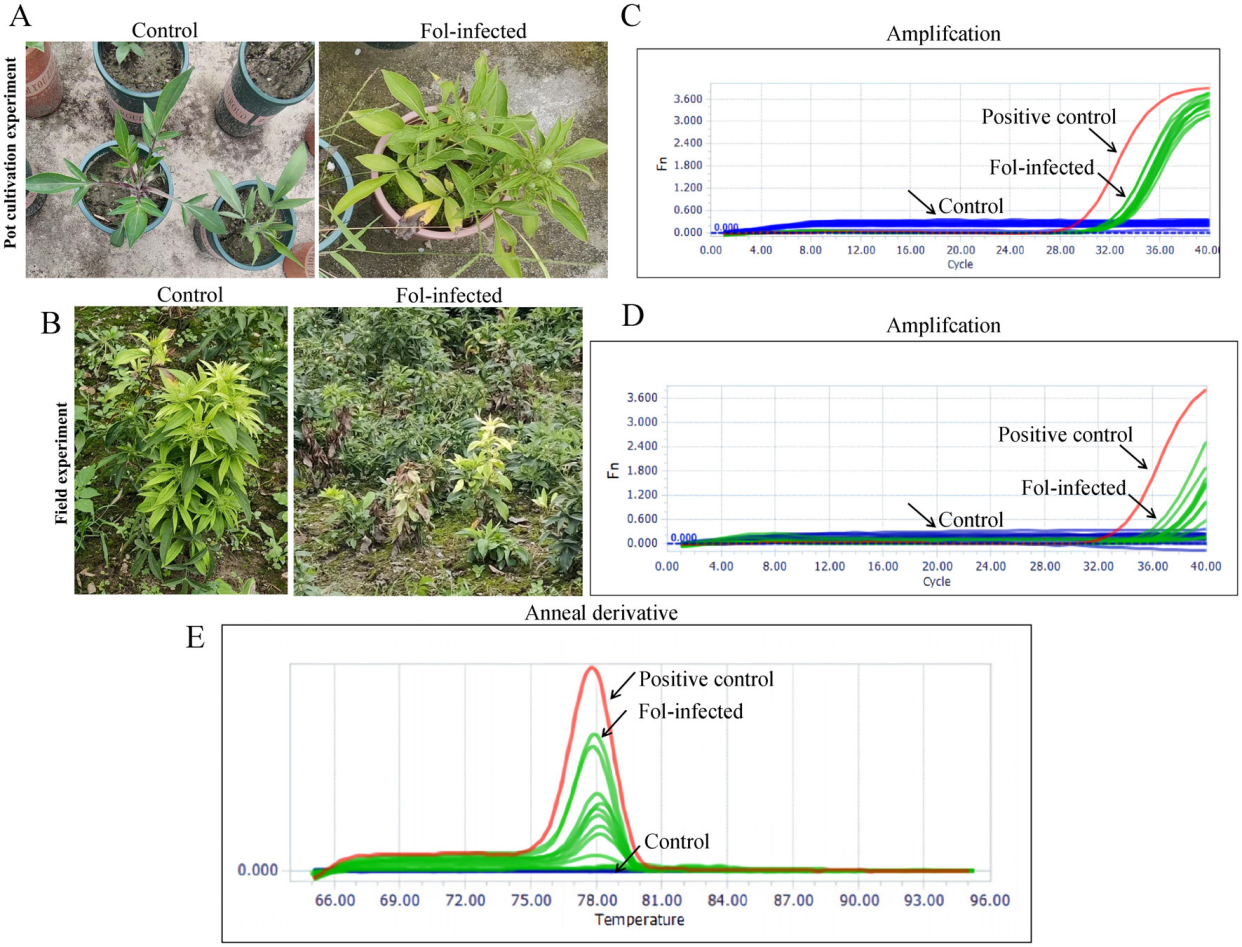
Validation of the practical samples. Representative images of Fol-infected AMK for 5 months from **(A)** Pot cultivation experiments and **(B)** field experiments. **(C)** Amplification profiles of SIX1 of the practical samples from Pot cultivation experiments and field experiments by using Fol one-step LAMP-CRISPR/Cas12b method, the. The recombinant plasmid was served as the positive control (PC), while the nuclease-free water used for NTC. **(D)** Amplification profiles of SIX1 of the practical samples from Pot cultivation experiments and field experiments by using qPCR assays. **(E)** Anneal derivative of SIX1 amplicons within qPCR assays, with an average anneal derivative of 78°C.

To validate the performance of the present reaction for practical samples, the Fol-infected AMK samples were collected from pot cultivation (n=6) and field cultivation (n=6) respectively. Moreover, the non Fol-infected AMK was used as the control (n=6) for pot cultivation (n=6) and field cultivation respectively. As shown in **Figures 8A, B**, all the Fol-infected AMK samples showed a typical morphological characteristic compared with that of the control. After extracting the nuclear acid samples, these 24 real samples were simultaneously detected by using method and PCR method. According to the amplification curve, all the 6 Fol-infected samples and 6 control samples from pot or field cultivation were identified by one-step LAMP-CRISPR/Cas12b and the PCR method (**Figures 8C, D**). Meanwhile, the anneal temperature of the PCR for SIX1 amplification was specificity at 78°C (**Figure 8E**). Moreover, the detection results by using the one-step LAMP CRISPR/Cas12b method were totally in agreement with that of the PCR-based method. The consistency of these two methods achieved 100% (24/24, **Table 3**).

**Table 3:** Comparison between performance of the Fol one-step LAMP-CRISPR/Cas12b method and qRCR.

	Fol one-step LAMP-CRISPR/Cas12b		qPCR		Sensitivity	Specificity	Consistency
	No. positive	No. negative	No. positive	No. negative			
Positive	12	0	12	0	100%	100%	100%
Negative	0	12	0	12			
Total	12	12	12	12			

## 4 Discussion

In China, there is a long history of AMK cultivation and application as a traditional Chinese medicine (Liu et al., 2022). Due to the outstanding performance in relieving multiple human diseases, maintaining a stable yield of AMK is critically important (Yu et al., 2023; Chen T et al., 2023; Chen F et al., 2023). As a perennial herb, the Fol-induced root rot during the long-term continuous cropping caused heavy losses for AMK cultivation. Therefore, an available detection method for Fol diagnosis is conducive to preventing the outbreak of root rot and save losses for AMK planter.

Commonly, the traditional approach for preventing Fol relied on chemical treatment by using fungicides or agrochemicals (Amini and Sidovich, 2010). However, the outcome is far away from satisfactory due to the contamination of soil and toxicity to beneficial organisms (Yu et al., 2017). The efficient diagnosis of crop diseases plays an important part in disease management. In the specific case of Fol, finding and eradicating any plants infected with Fol at an earlier cultivation stage, and avoiding the cultivation of host crops in Fol-contaminated areas are both helpful for reducing the economic impacts of Fol. Therefore, a rapid and specific method of detecting Fol has become a crucial strategy for the effective monitoring and control of the pathogen (Chang et al., 2022).

Recently, CRISPR-based assay for plant pathogen diagnostics was widely reported, such as RPA/Cas12a-based detection of *Aphelenchoides besseyi* in rice (Zhang et al., 2022), *Fusarium asiaticum* in maize and wheat (Zhang et al., 2023), *Heterodera avenae* and *Heterodera filipjevi* in cereal crops (Shao et al., 2023). To date, in the field of plant pathology detection, all CRISPR-based detection are two individual reaction steps, including pre-placed on the tube lid or all-in-one chip lateral flow assay (Lei et al., 2022). However, all these methods suffered from complex manual operations and cross-contamination.

In the current research, we firstly developed a one-step Fol detection via combining LAMP assay and CRISPR/Cas12b reaction in one single tube. The cross-contamination was totally avoided. The entire detection can be finished within 45 min at a constant temperature of 60°C. There was no additional complex operation and friendly for fresh users. Moreover, the LoD of the one-step LAMP CRISPR/Cas12b platform reached 88.9 copies per reaction and no false-positive results were observed by using interfere samples. Therefore, the one-step LAMP CRISPR/Cas12b platform showed super sensitivity and specificity for Fol detection, outputting a reliable result.

Nowadays, PCR-based methods have widely been used for pathogens detection and function as the gold standard (Lievens et al., 2009; Pothiraj et al., 2022). Here, the one-step LAMP CRISPR/Cas12b presented a well performance for detecting Fol-infected AMK samples, yielding a 100% consistency with the PCR-based method. Therefore, the efficiency of the one-step LAMP CRISPR/Cas12b for Fol detection was acceptable. Moreover, there was no additional requirement as that of PCR-based method for one-step LAMP CRISPR/Cas12b platform. Importantly, the key components can be pre-prepared by lyophilization. Therefore, our present findings not only established a platform for Fol detection in AMK but also is a potentially efficient procedure for extending to resource-limited regions and on-site detection.

## 5 Conclusion

In conclusion, we first developed a one-step LAMP-CRISPR/Cas12b method for Fol detection in AMK, attaining the advantages of rapid, simple, and accurate. Our present findings not only established a platform for Fol detection but also highlighted the potential value of CRISPR-based methods for other phytopathogen detection.

## Data Availability

The original contributions presented in the study are included in the article/supplementary material. Further inquiries can be directed to the corresponding author.
